# Transcriptome reprogramming during developmental switching in *Physarum polycephalum* involves extensive remodeling of intracellular signaling networks

**DOI:** 10.1038/s41598-017-12250-5

**Published:** 2017-09-26

**Authors:** Gernot Glöckner, Wolfgang Marwan

**Affiliations:** 1 Institute for Biochemistry I, Medical Faculty, University of Cologne, Joseph-Stelzmann-Straße 52, 50931 Köln, Germany; 2 Magdeburg Centre for Systems Biology (MaCS) and Institute for Biology, Otto von Guericke University, Pfälzerstrasse 5, 39106 Magdeburg, Germany

## Abstract

Activation of a phytochrome photoreceptor triggers a program of *Physarum polycephalum* plasmodial cell differentiation through which a mitotic multinucleate protoplasmic mass synchronously develops into haploid spores formed by meiosis and rearrangement of cellular components. We have performed a transcriptome-wide RNAseq study of cellular reprogramming and developmental switching. RNAseq analysis revealed extensive remodeling of intracellular signaling and regulation in switching the expression of sets of genes encoding transcription factors, kinases, phosphatases, signal transduction proteins, RNA-binding proteins, ubiquitin ligases, regulators of the mitotic and meiotic cell cycle *etc*. in conjunction with the regulation of genes encoding metabolic enzymes and cytoskeletal proteins. About 15% of the differentially expressed genes shared similarity with members of the evolutionary conserved set of core developmental genes of social amoebae. Differential expression of genes encoding regulators that act at the transcriptional, translational, and post-translational level indicates the establishment of a new state of cellular function and reveals evolutionary deeply conserved molecular changes involved in cellular reprogramming and differentiation in a prototypical eukaryote.

## Introduction

Cell differentiation, the phenomenon of forming cell types that are specialized in structure and function is enabled by differential gene expression. Complex differentiation is especially a hallmark of the eukaryotes, while prokaryotes normally have only relatively simple differential cell types (e.g. Nitrate-producing cells in filamentous cyanobacteria^1^). Multicellular organisms, animals, plants, and representatives of the polyphyletic group of thallophytes are composed of different cell types of specific functions as building blocks of a structurally organized body. Across kingdoms, in many unicellular eukaryotes, specialized cell types instead of shaping a body or thallus, occur in temporal order in the course of a so-called life- or developmental cycle which often involves the meiotic recombination of the genome with stages of gametogenesis, mating, and the formation of dormant cell types^2^.

**Figure 1:**
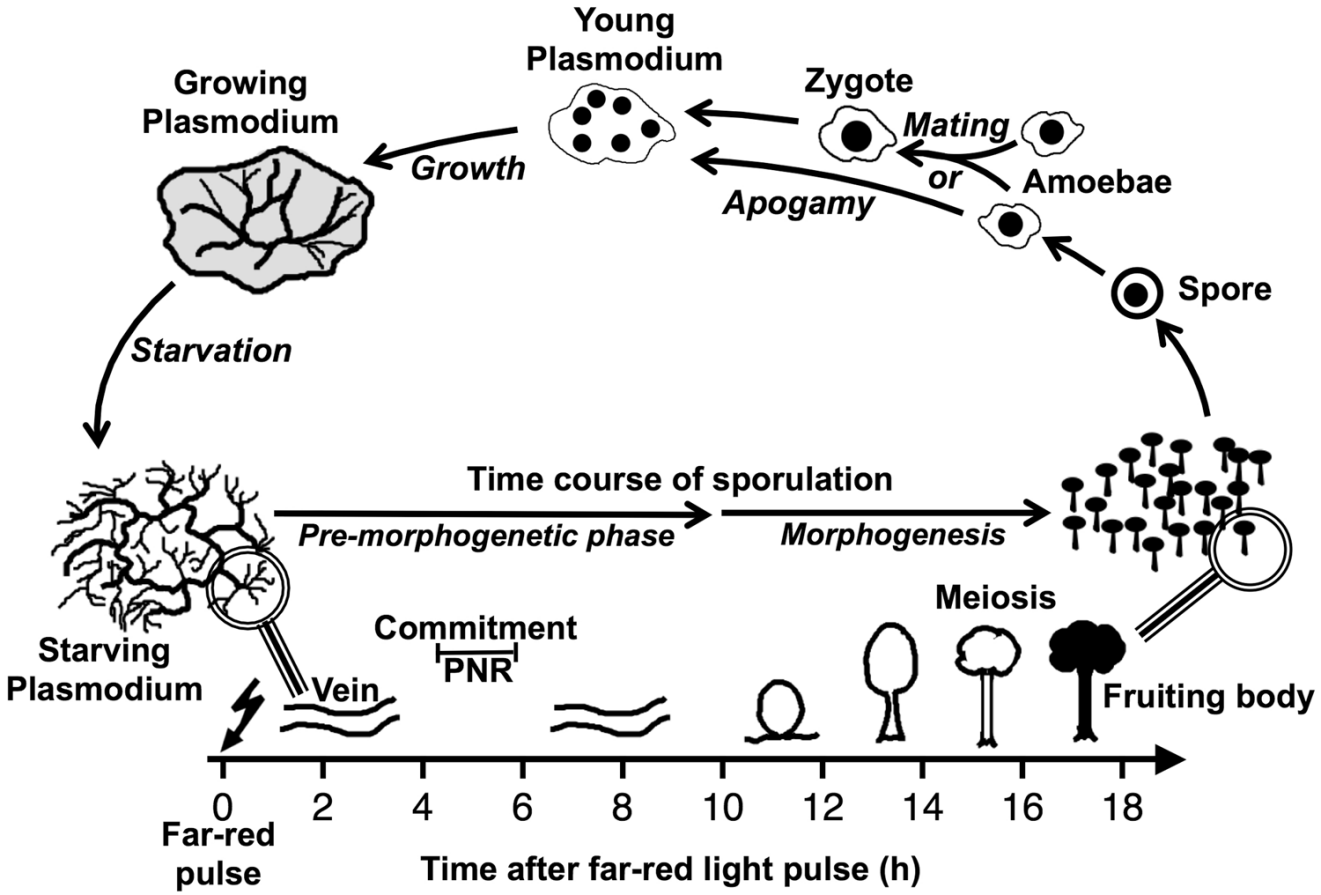
Time-course of sporulation triggered by far-red light as displayed in the context of the simplified life cycle of *Physarum polycephalum*. Sporulation of a starving, competent, multinucleate (macro-) plasmodium can be experimentally induced by a brief pulse of far-red light which is sensed by a phytochrome photoreceptor (ref.^21^ and references therein). After the inductive light pulse there is a pre-morphogenetic phase without any visible changes in the plasmodial morphology. By crossing the commitment point (point of no return, PNR), the plasmodium loses both, its unlimited replicative potential (seen as the ablility to grow on nutrient agar) and the alternative option of developing into a drought-resistant, dormant sclerotium (not shown). Morphogenesis then starts at about 9 to 10 hours after induction when the plasmodial strands wind up and subsequently break up into small nodular structures (nodulation stage). Each nodule culminates and differentiates into a melanized fruiting body. Sporangiophores (stalkes) and the sporangial wall, the peridium, are formed by extracellular material^3^ and not by specialized cell types as in social amoebae. By meiosis and cleavage of the multinucleate protoplasmic mass, haploid, mononucleate spores are formed and can lateron be released from the fruiting body. Under favourable conditions, a spore can germinate to release a haploid, mononucleate amoeba which feeds on bacteria and propagates by mitotic cell division (not shown). At high population density, and depending on its genotype, an amoeba can either directly develop into a haploid, multinuclear plasmodium (apogamy) or alternatively mate with another amoeba that carries a compatible combination of mating type alleles to form a diploid zygote that develops into a multinucleate plasmodium^34^. Following growth and starvation, the plasmodium, no matter whether haploid or diploid, develops its competence for the induction of sporulation by visible light or heat shock^4,11^. Meiosis of a small subpopulation of diploid nuclei that developed by endoreduplication of haploid nuclei leads to the formation of a small fraction of fertile spores upon sporulation of a haploid plasmodium^35^. Haploid and diploid developmental cycles both occur in wild type isolates of myxomycetes, suggesting that both are natural, physiological processes^36^. For the sake of simplicity, formation of flagellates or cysts from amoebae and the development of a plasmodium into a sclerotium have been omitted from this scheme.

Pronounced cell differentiation processes and even multicellular development in response to environmental conditions are found in the mycetozoan branch of the amoebozoa group of organisms: *Dictyostelia* proceed from unicellular amoebae that aggregate into a multicellular slug to develop a fruiting body composed of different cell types while *Physarum polycephalum* (a member of the *Myxogastria*) proceeds through a branched developmental cycle in which the different cell types differentiate in temporal order^3,4^ (Fig. 1). Sporulation of the *Physarum polycephalum* plasmodium served as a model for cell differentiation and development as it displays basic phenomena of developmental biology: competence, induction, commitment (determination), differentiation, meiosis, and gametogenesis^5–7^. Sporulation of a starved, plasmodium can be experimentally induced by a brief pulse of visible light^6,8–10^. Approximately four to six hours after an inductive light stimulus, the plasmodium crosses the point of no return, irreversibly loses its unlimited replicative potential^6,8,11^, and forms fruiting bodies several hours later (Fig. 1). It has been shown that commitment and sporulation are associated with extensive alterations of the gene expression pattern^12–15^ and that differential gene regulation already occurs before the commitment point^16,17^.

Comparative analysis of developmentally essential and developmentally upregulated genes across Dictyostelid species from different evolutionary branches^18–20^, suggested that the transition to multicellularity required novel signals and sensors rather than novel signaling mechanisms. Within this context, switching of the *Physarum* plasmodium to sporulation is particularly interesting: (1) *P*. *polycephalum* is evolutionary distant from the *Dictyostelids* while both Amoebozoa, due to their position within the phylogenetic tree share a common ancestor^21^ and may help to define prototypical core regulatory mechanisms. (2) Since sporulation of the *Physarum* plasmodium is holocarpic, it allows to study the switch of a single, synchronously developing multinucleate cell from a proliferating to a differentiating state and hence the transition between two cell types in a quantitative and time-resolved manner^16,17,22^. This may provide additional and eventually complementing mechanistic information on Amoebozoan differentiation processes. We therefore performed a transcriptome-wide analysis of differential gene expression during commitment and sporulation of plasmodial cells and found that the transcriptional changes reveal extensive remodeling of intracellular signaling networks.

## Results

### Reference transcriptome and sequence similarity searches

The *P*. *polycephalum* genome proved difficult to assemble. Therefore, the analysis of it relied heavily on the reference transcriptome data, which were constructed from all available RNAseq data^21^. In favour of the best possible assignment of RNAseq sequence reads to corresponding genes and the subsequent quantification of the transcript abundances, we have revised the reference transcriptome. To construct this new version used here we additionally joined neighbouring transcripts if they i) overlapped, or ii) together represented genes indicated by BLAST hits against data base entries from other organisms. Furthermore, only one version of obviously alternatively spliced transcripts (indicated by identical sequences of more than 100 bases) was retained. The new version is accessible through http://www.physarum-blast.ovgu.de. The sequences of the revised reference transcriptome were queried for similarity to sequences in the UniProt database, for domains annotated in the pfam and Prosite databases (Supplementary Table 1), and for similarity to *Dictyostelium discoideum* genes (Supplementary Table 2; see the electronic version of this article for Supplementary Information with a description of all Supplementary Tables). The revised version of the reference transcriptome contained 28139 sequences, thus reducing the original 31770 reference transcripts significantly. 47% of these shared significant similarity to sequences from the UniProt database. For 15197 of all transcripts and for 3018 of the transcripts without UniProt annotation, sequence similarities to one or more known protein domains were detected. The query results are given as supplementary information.

### Analysis of developmental switching

**Figure 2:**
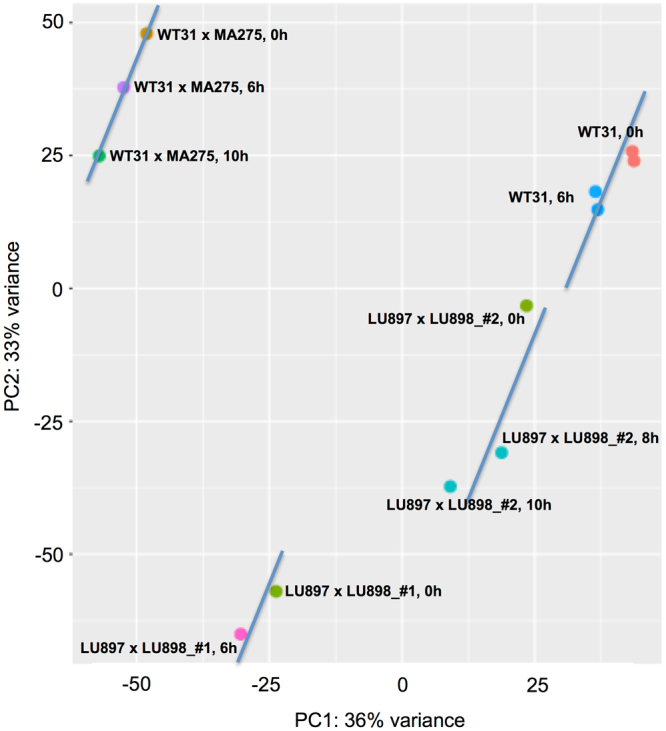
Gene expression patterns in samples of light-induced plasmodia taken at different time intervals after a far-red light stimulus pulse (6 h, 8 h, 10 h) and dark controls (referred to as 0 h samples) were compared by principle component analysis. Data points representing the samples taken during the same experiment (see Table 1 for details) were connected by a straight line to guide the eye.

Cells competent for sporulation were exposed to a brief pulse of light to induce the differentiation process. Controls (see Methods) ensured that no spontaneous sporulation occurred in the tested samples. Table 1 lists all different plasmodia and time points from which the samples for RNA isolation were taken. A principal component analysis (PCA) of the individual samples showed that strain specific effects and potential unintended minor variations in the experimental conditions resulted in clustering of strain data (Fig. 2). However, the differentiation induced effects on the transcriptomes seem to be similar in all experiments as indicated by the similar shifts in positions in the PCA analysis. Thus, the different samples provide biological replicates and the individual sample counts for different plasmodia could be combined to minimize these strain-specific effects on further downstream analyses.

**Table 1:** Samples used for RNAseq analysis. See Methods for experimental conditions.

Experiment	Strain	Starved for	Far-red (FR)	Dark control or time after FR	Sporulated plasmodia	Matched to give dataset			Seqcount
#1	LU897 × LU898	6d	—	DC	0 of 1	0–6 h		0–10 h	5915413
	LU897 × LU898	6d	40 min	6 h	1 of 1	0–6 h	6–10 h		5678394
#2	LU897 × LU898	6d	—	DC	0 of 1	0–6 h		0–10 h	15242846
	LU897 × LU898	6d	40 min	8 h	1 of 1		6–10 h	0–10 h	40986624
	LU897 × LU898	6d	40 min	10.5 h	1 of 1		6–10 h	0–10 h	23138471
#3	WT31	6d	—	DC	0 of 1	0–6 h		0–10 h	19941711
	WT31	6d	—	DC	0 of 1	0–6 h		0–10 h	19499417
	WT31	6d	30 min	6.5 h	1 of 1	0–6 h	6–10 h		18279213
	WT31	6d	30 min	6.5 h	1 of 1	0–6 h	6–10 h		19346090
#3	WT31 × MA275	7d	—	DC	0 of 5	0–6 h		0–10 h	36374249
	WT31 × MA275	7d	40 min	6 h	4 of 5	0–6 h	6–10 h		37574067
	WT31 × MA275	7d	40 min	8 h	5 of 5		6–10 h	0–10 h	38406990

Expression level as defined by read counts was calculated after alignment of sequence reads to the model transcripts of the revised version of the *P*. *polycephalum* reference transcriptome using bowtie2. The data of different wild-type strains, haploid and diploid, were taken and, at a later stage of the analysis, combined in order to minimize strain- or genotype-specific effects (see below).

### Identification and characterization of differentially regulated genes

In order to determine significant overall light-induced changes, transcript counts were evaluated with the help of DESeq. 2 package^23,24^. Each of the three time points (0 h, 6 h and 10 h) had biological replicates, ensuring that only the most robust transcriptomic changes would be captured by our analysis. We used a p-value of <0.025 to define significantly up- or downregulated genes between those three time points. Since we were interested in analysing discernable transcriptional changes during transition to sporulation, each transcript was analysed in respect to the time interval where its maximal differential regulation occurred.

**Figure 3:**
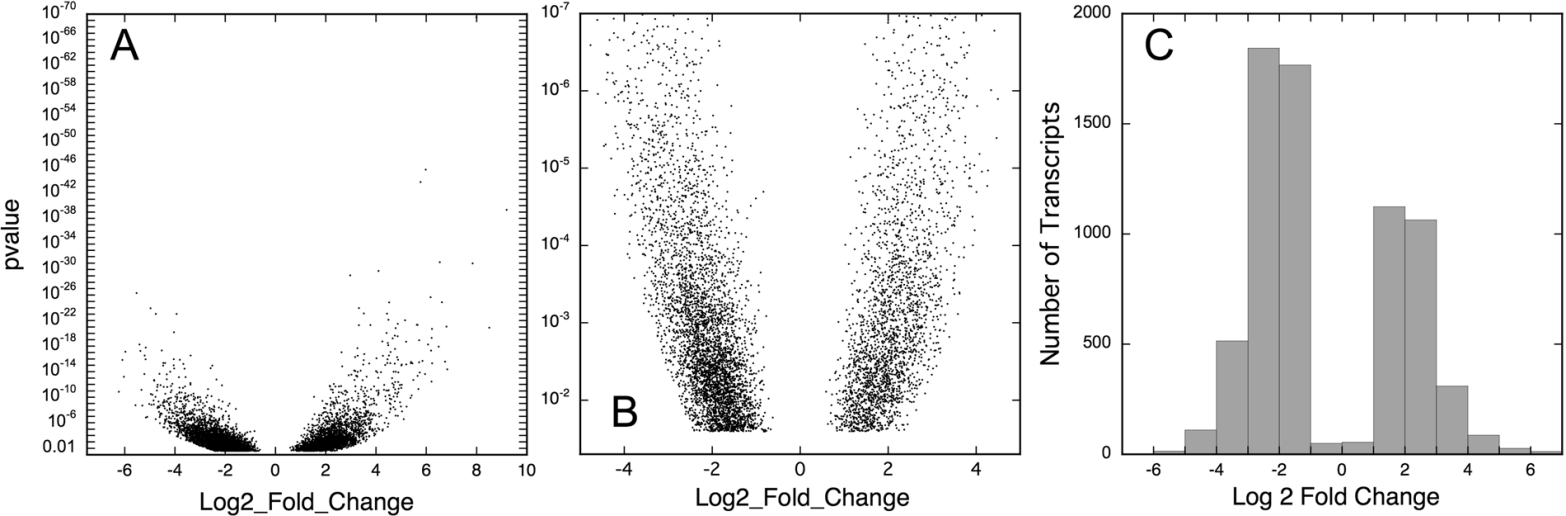
Values of Log2 fold changes in read counts as obtained for the maximal significant change that occurred for each transcript of the reference transcriptome. Log2 fold changes were determined by comparing the expression patterns of two states, xh *vs*. y h, for each of the three data sets (0–6 h, 6–10 h, 0–10 h), respectively as described in Materials and Methods and the maximal change that was significant (*p* < 0.025) was determined for each transcript. (**A**) The combination of the maximal Log2 fold change and its pvalue is displayed for each individual transcript of the reference transcriptome. Accordingly, each transcript gave one data point only. (**B**) Section of (**A**) zoomed out for pvalues ≥ 10^−7^. (**C**) Frequency distribution of the number of transcripts that showed the highest significant Log2 fold change whithin one of the three datasets (0–6 h, 6–10 h, 0–10 h). As in (**A**) each individual transcript of the reference transcriptome is represented only once, provided it changed significantly (*p* < 0.025).

The DESeq. 2 algorithms identified 6985 transcripts that according to our criteria were differentially regulated in at least one of the three datasets with log2-fold changes ranging between −6.23 to −0.62 and 0.59 to 9.19 corresponding to a change in the expression strength between 1.5 to 75-fold for down-regulated and 1.5 to 584-fold for up-regulated genes (Fig. 3; see Supplementary Table 3 for the complete set of significantly differentially regulated transcripts).

**Figure 4:**
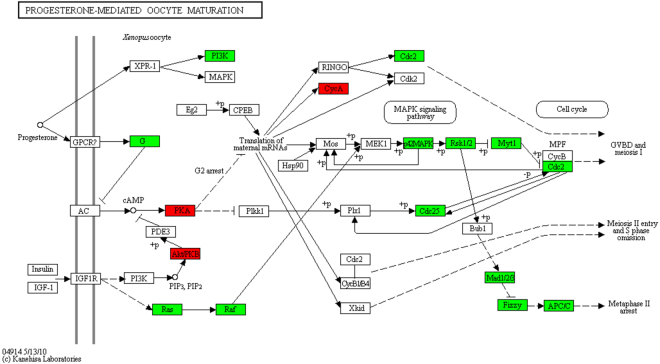
Differentially expressed transcripts encoding *P*. *polycephalum* homologs of proteins involved in progesterone-mediated oocyte maturation. The figure shows screenshots taken from the KEGG database^25–27^. Proteins encoded by up-regulated genes are shown in green, down-regulated ones in red. With kind permission by the Kanehisa Laboratories.

Approximately 50% (3556) of the 6985 transcripts found to be differentially regulated, shared similarity with proteins in the UniProt database (Table 2) and 60% of this subgroup could be assigned a KEGG annotation (not shown). Out of the KEGG annotated transcripts, 75% corresponding to 1594 transcripts could be assigned to KEGG annotated pathways. Furthermore, out of 1875 transcripts with UniProt annotation that displayed at least 4-fold changes (up or down), 813 could be assigned to KEGG annotated pathways. Members of the most abundant group of transcripts similar to genes with KEGG annotation (151) encoded enzymes of metabolic pathways. Others are invoked in the MAP-kinase pathway, cell cycle, oocyte meiosis or homologous recombination as expected for a plasmodial cell, which resides in the vegetative phase and which is committed to sporulation. If we omit transcripts with weaker differential expression we see that transcripts with more than 6-fold changes encoded protein homologs known to control proliferation and differentiation like SOS, Ras, Ral, PI3K, PTEN, Akt/PKB, PKA, Rho and Arf being involved in Phospholipase D signaling and/or in other core signaling pathways. Figure 4 shows pathways of oocyte maturation and oocyte meiosis as retrieved from the KEGG database^25–27^. Proteins are highlighted with a colour code according to the mode of the differential regulation of the homolog-encoding mRNAs in *P*. *polycephalum*.

**Table 2:** Transcripts with significant up- or down-regulation as detected in the three datasets. Duplicate social amoebae gene indentifiers were not considered for the counts.

Regulation	Max change at	All	Hits without UniProt Annotation	Hits with UniProt Annotation	Social Amoebae DR Orthologs*	% Physarum/Social Amoebae with UniProt
down	0–6 h	187	65	122	nd	
	6–10 h	674	428	246	nd	
	0–10 h	3440	1847	1593	nd	
	total	4301	2340	1961		
up	0–6 h	201	99	102	17	16.7
	6–10 h	475	168	307	42	13.7
	0–10 h	2009	808	1201	174	14.5
	total	2685	1075	1610	233	14.5
up & down		6986	3415	3571	—	
					*Duplicates eliminated

**Figure 5:**
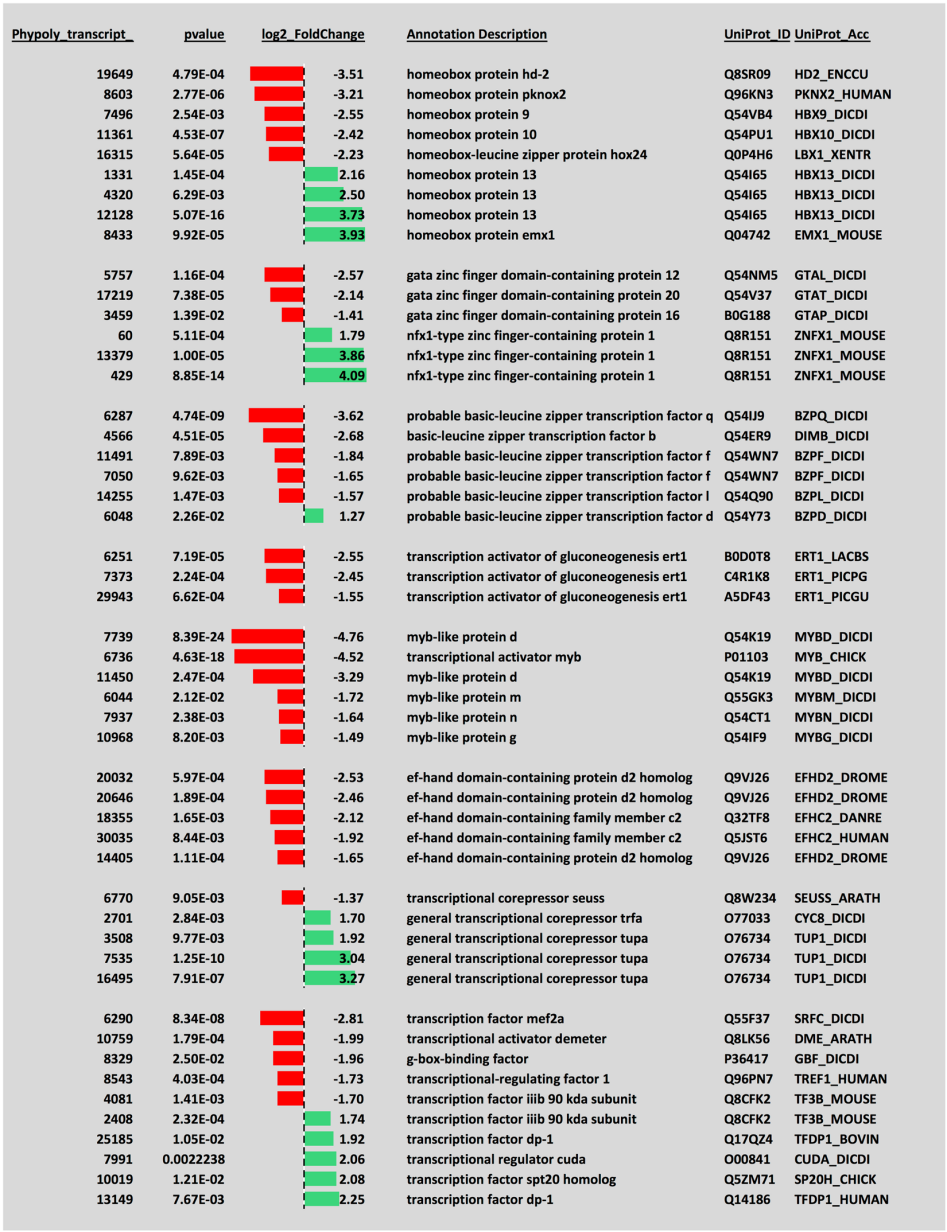
Differential expression of transcripts encoding transcription factor homologous proteins. The 0–10 h dataset was inspected for differentially regulated transcripts with homology to known transcription factors according to their annotation description with UniProt_ID and UniProt_Acc within the UniProt database. The p-values indicate the significance of differential regulation. Log2 fold changes are plotted for transcripts that are down-regulated (red) or up-regulated (green) in response to the sporulation-inducing far-red light stimulus.

To obtain a more detailed view on the molecules involved in reprogramming, differentially regulated transcripts were grouped manually according to their UniProt descriptions and functional assignments (Supplementary Table 4). Clearly, developmental switching was associated with a change in expression of the prevailing classes of transcription factors. GATA-, basic-leucine zipper, and myb-like transcription factors along with transcription activators of gluconeogenesis were down-regulated while, among others, nfx1-type zinc finger-containing proteins, the transcriptional regulator *cudA*, isoforms of the general transcriptional corepressor *tupA*, and isoforms of the transcription factor Dp-1 were up-regulated in a highly significant manner (Fig. 5). Approximately half of the differentially expressed transcription factor-encoding genes had highest similarity to *Dictyostelium discoideum* genes while the others were most similar to homologs from higher animals, *Arabidopsis* or yeasts (Fig. 5). In contrast, specific homeobox proteins were regulated antagonistically, some were down- and some were up-regulated indicating a switch to required homeobox transcription factors. Differential expression and switching between specific classes of transcription factors predicts extensive transcriptional reprogramming during sporulation and development of meiotic, dormant spores from a vegetative, mitotic, multinucleate plasmodium.

**Figure 6:**
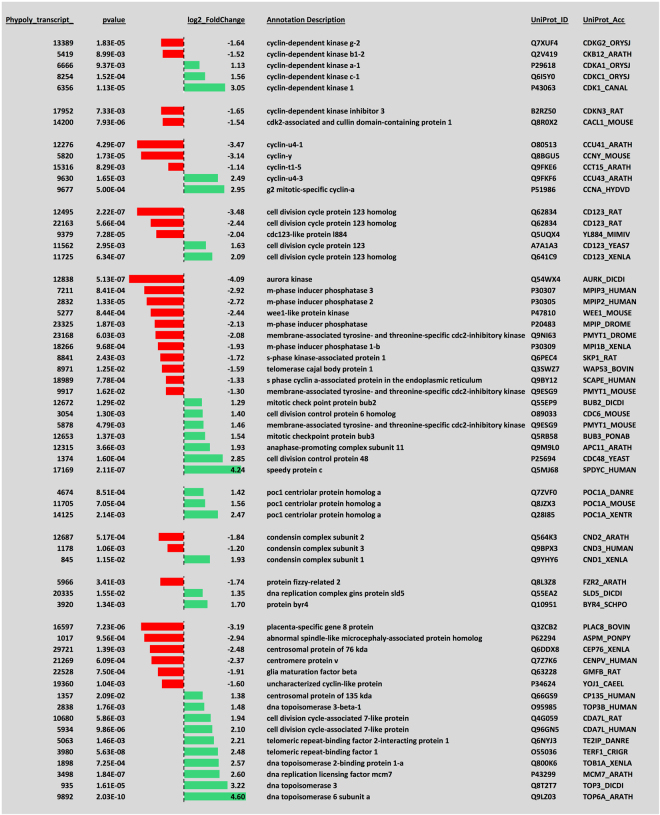
Differential expression of transcripts encoding regulators and other components involved in the eukaryotic cell cycle. The panel was generated as described in the legend to Fig. 5.

**Figure 7:**
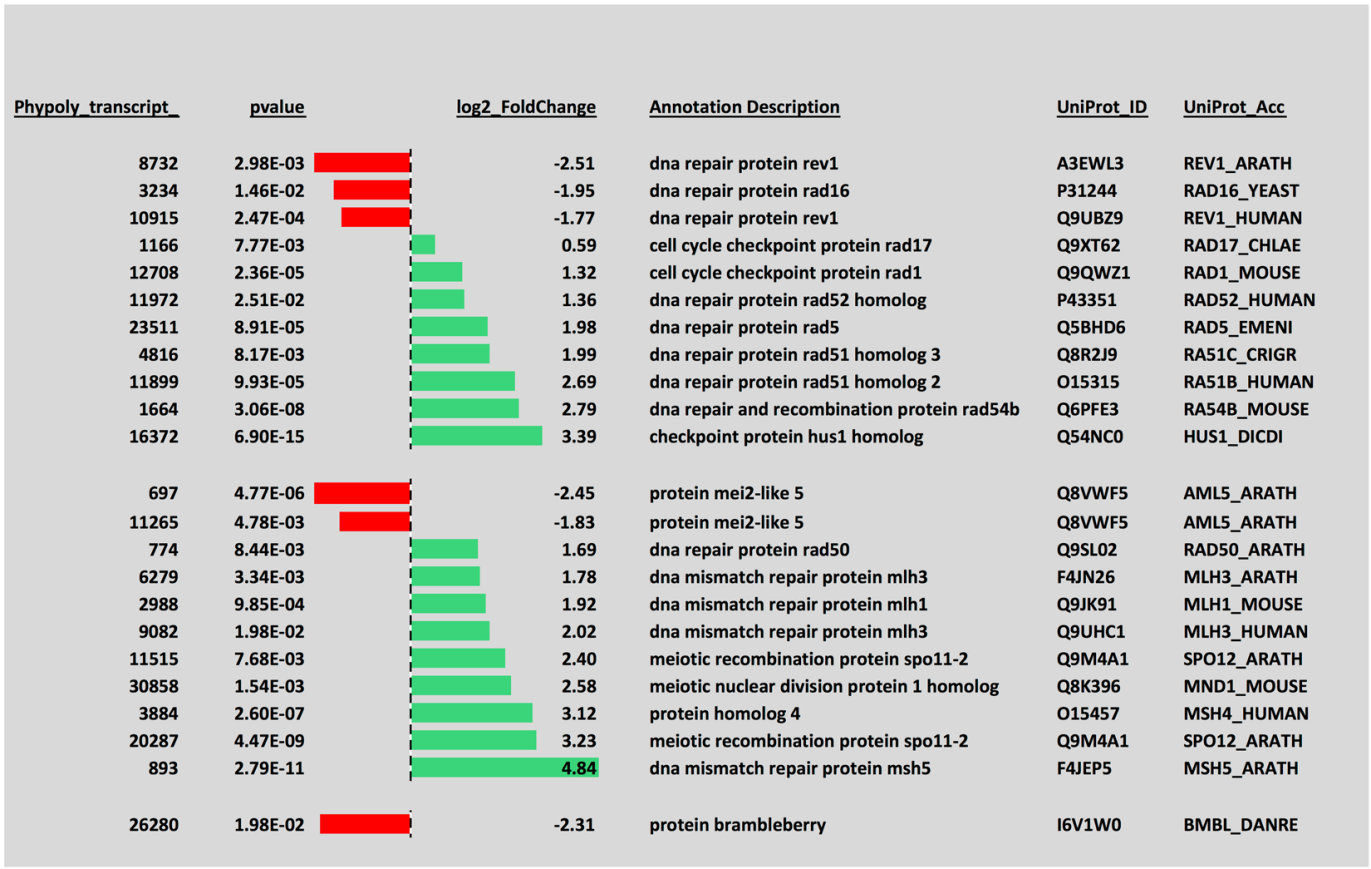
Differential expression of transcripts encoding proteins involved in DNA repair or meiotic recombination. The panel was generated as described in the legend to Fig. 5.

**Figure 8:**
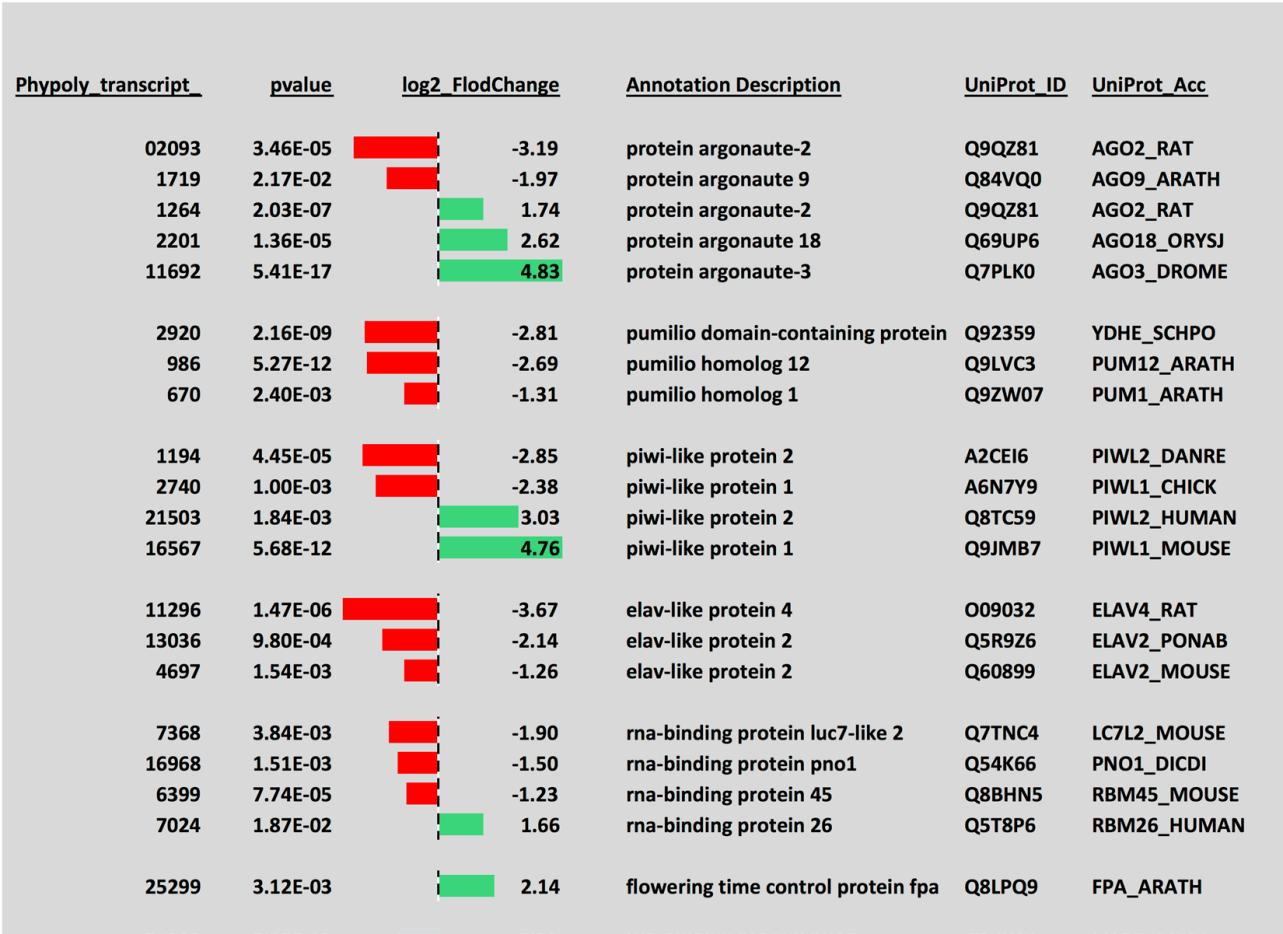
Differential expression of of transcripts encoding RNA-binding proteins. The panel was generated as described in the legend to Fig. 5.

Transition from a vegetative, mitotic plasmodium to meiotic spores certainly requires mechanisms for the differential regulation of mitotic and meiotic cell cycles. Indeed, transcripts encoding a considerable number of cell cycle regulators, DNA repair proteins, and proteins involved in meiotic recombination were differentially regulated (Figs 6 and 7), indicating major rearrangements within the cell cycle control machinery. In line with cell type switching, the equipment with RNA-binding proteins also changed markedly. Transcripts encoding pumilio and ELAV-like proteins were downregulated while the expression of piwi and argonaute proteins switched between homologs suggesting that the differential regulation of different sets of genes at the translational level occurs in the course of cell type transition (Fig. 8).

Extensive reprogramming was also obvious for many different types of commonly known, important cellular regulators. The differential expression of a high number of serine-threonine kinases, of hybrid signal transduction histidine kinases, and of various types of phosphatases suggests the establishment of a new functional state of the cell with globally altered specificity in signaling. The extensive differential regulation of protein degradation by ubiquitinylation and sumoylation is suggested by the numerous E3 ubiquitin-protein ligases that are differentially regulated (Supplementary Table 4).

### Comparison to the core set of developmentally regulated genes in social amoebae

Previously, sets of genes important for development in social amoebae were defined by either being up-regulated in *Dictyostelium discoideum*, *D*. *lacteum*, *D*. *fasciculatum*, and in *Polysphondylium pallidum* ^20^ or by exhibiting a developmental defect after knockout^18^ revealing a core set of 1167 genes with evolutionary conserved developmental regulation in social amoebae. In order to identify orthologs or paralogs, the *P*. *polycephalum* transcriptome was BLASTed against this set. A total of 1007 *D*. *discoideum* genes (86%) out of the social amoebae core set gave 1013 BLAST hits to 739 *P*. *polycephalum* genes. 681 *D*. *discoideum* genes shared similarity with *P*. *polycephalum* transcripts that were not differentially regulated. The remaining 332 *D*. *discoideum* genes gave BLAST hits to 233 differentially regulated *P*. *polycephalum* transcripts. Although the social amoebae core set comprised exclusively up-regulated genes, 199 BLAST hits to up-regulated and 133 to down-regulated *P*. *polycephalum* transcripts were found, indicating an evolutionary change of transcription regulation. Details with respect to the time intervals of differential regulation are given in Table 2. Strongest up- and down-regulated genes out of this list define candidates of a core set of genes the differential regulation of which is deeply conserved in Amoebozoa evolution. These strongest differentially regulated genes display a strong bias towards roles in signal transduction or gene regulation (Supplementary Table 5). As their differential regulation is associated with commitment and differentiation, at least some of the encoded proteins presumably play a role in the core regulatory network of cell differentiation.

In summary, sporulation of *P*. *polycephalum* plasmodial cells was found to be associated with extensive reprogramming of gene expression including the differential expression of transcription factor-encoding genes. In addition, the differential expression of genes encoding RNA-binding proteins and enzymes catalyzing protein phosphorylation or ubiquitinylation suggests extensive differential regulation at the translational and post-translational level, respectively. Reprogramming and the switching from mitotic to meiotic competence was associated with the differential expression of cell cycle regulators and of homologs of core regulators controling proliferation and differentiation in mammalian cells.

## Discussion

Based on an improved reference transcriptome, we have analysed the transcriptional changes associated with developmental switching of the *P*. *polycephalum* plasmodium to sporulation. Samples of total RNA were taken at different time points after triggering sporulation by activating a phytochrome photoreceptor with a brief far-red light pulse^21,28,29^. To minimize the detection of genotypic and strain-specific effects on differential gene expression, we combined RNAseq datasets obtained from wild type strains that differed in genotype and ploidity. Pooling of the data allowed the transcriptome-wide detection of statistically significant overall changes that occurred during commitment and sporulation. Accordingly, we deliberately did not perform a detailed evaluation and interpretation of temporal patterns of gene expression because there are differences between individual plasmodial cells^17,30^. Consequently, the x-fold changes in the abundances of certain transcripts may be underestimated as compared to the maximal changes of certain genes that were detected in individual cells^17^.

Besides the differential regulation of metabolic genes, significant changes encompassed virtually all types of molecules that are involved in cellular signaling and regulation. Simultaneous up- and down-regulation of genes encoding similar types of proteins (e.g. kinases) was the rule rather than the exception, indicating replacements rather than shut down or increase of the corresponding reactions. This indicates an extensive remodeling of the intracellular signaling networks by groupwise replacement of many types of mediators and revealing the establishment of a new state of cellular differentiation. The differential regulation of transcription factors, of RNA-binding proteins, of large numbers of protein-modifying enzymes, and of ubiquitin ligases reflects that differential regulation occurs at the transcriptional, translational, and post-translational level. Differential regulation of cyclins, cyclin-dependent kinases, and other cell cycle regulators is noteworthy and might help in establishing links between cell cycle regulation and differentiation. Starving plasmodia that are competent for the induction of sporulation are still able to proceed through mitotic cycles^6^ and resume growth when fed with glucose. In contrast, sporulation involves meiosis to generate the haploid nuclei of mononucleate spores. As meiosis, also in *P*. *polycephalum*, can be considered as an essential step in gametogenesis, it is not surprising that quite a number of genes that are up-regulated share close similarity to genes encoding regulators of oocyte maturation and oocycte meiosis. The similarities of differentially regulated genes to mitotic and meiotic regulators suggest that remodeling of the cell cycle control network is associated with the developmental decision to sporulate. To gain mechanistic insight however, further experimental analyses are necessary.

Clearly indicative for the establishment of a new state of cellular differentiation is the exchange of myb-like, basic leucine zipper and gata zinc finger transcription factors by nfx1-type zinc finger transcription factors as well as the antagonistic regulation of a set of homeobox proteins all of which are seen at the level of encoding RNAs.

The comparison of *P*. *polycephalum* transcripts to the core set of developmentally up-regulated genes in social amoebae^20^ performed in this study may help to consolidate the set of genes or functions that are evolutionary deeply conserved in regulating differentiation in Amoebozoa and possibly in other eukaryotic cells. Genes switched off during the development of social amoebae (aggregation, mound formation, early and late fruiting body development) encode predominantly proteins involved in growth and metabolism of vegetative cells, including ribosomal proteins *etc*. but rarely signal transduction or gene regulatory proteins^20^. In contrast, many proteins with roles in cellular regulation and gene expression control are up-regulated during development and because of the evolutionary conservation of their up-regulation among distantly related species these genes were assigned to a core set that is associated with the establishment of multicellularity in social amoebae and accordingly at least in part with cell differentiation^20^.

Most (87%) of the 1167 genes of this core set share similarity to transcripts expressed in *P*. *polycephalum* plasmodia but hardly 15% of these genes correspond to transcripts that are differentially regulated during and after the commitment to sporulation of *P*. *polycephalum*. This discrepancy might partly be due to the different developmental programs of social amoebae and myxomycetes. In social amoebae, development of the fruiting body involves the differentiation of amoebae into different cell types during mound formation and culmination. It also involves the formation of multicellular structures in which the different cell types have different function and cells of different types are specifically positioned relative to each other. In *P*. *polycephalum* and other myxomycetes however, sporulation of the plasmodium involves the transition between two cell types only and not the formation of multicellular aggregates like in social amoebae.

Another possible reason why hardly 15% of the social amoebael core developmental genes were found to be differentially regulated in *P*. *polycephalum* might be that we were looking at commitment and the early phase of development. At approximately 10 h after light induction morphogenesis starts by entering the nodulation stage while culmination and formation of the mature fruiting bodies occurs during the subsequent eight hours.

Homologs of the social amoebae core developmental genes consisted of transcripts that were up- (60%) or down-regulated (40%) in *P*. *polycephalum*. The members of both groups of these genes (up- or down-regulated, respectively), as predicted by sequence similarity, are predominantly involved in cellular signaling and gene regulation. In social amoebae, these core developmental genes are up-regulated during development, while down-regulated genes are mainly house keeping genes required for cell growth and biosyntheses. This difference in regulation suggests that although evolutionary conserved core developmental functions are shared between dictyostelids and myxomycetales, the orchestration of the regulatory interactions may be different due to the clearly diverse developmental programs in the two groups of organisms.

The study presented here reveals the transcriptome-wide changes associated with the sporulation of plasmodial cells. The differentially regulated genes that were identified provide a valuable resource for further studies on the regulatory control of cell differentiation at the level of individual *P*. *polycephalum* plasmodial cells.

## Methods

### Growth and preparation of sporulation-competent plasmodia

Plasmodia were grown as a microplasmodial suspension culture for four days at 24 °C in 3 L of growth medium^31^ in a 5 L fermentor (Minifors, Infors HT, Bottmingen, Switzerland) inoculated with 2% of a 3.5 days old shaken culture, supplied with 1 L of air per minute, and stirred at 250 rpm with a marine propeller. Microplasmodia were harvested, washed twice with salt medium, and applied to starvation agar plates (9 cm diameter) with niacin and niacinamide^8^ as described^22^. A ring of 1 g of cell paste (fresh weight) was applied to the centre of each plate with the help of a motor-driven 50-mL syringe coupled to an in-house built automatic device for rotating the agar plate around its axis. Plates were incubated for 6 to 7 days at 22 °C in complete darkness. During this starvation period, one multinucleate sporulation-competent macroplasmodium developed on each plate. The incubation temperature is critical in order to avoid unwanted spontaneous sporulation. Sporulation of competent plasmodia was induced by a far-red light pulse (≥700 nm, 13 W/m^2^; see Table 1 for pulse lengths), generated by Concentra Weißlicht lamps (Osram, Munich, Germany) and passed through an Orange 478 combined with a Blue 627 plexiglass filter (Röhm, Darmstadt, Germany). After irradiation, plasmodia were returned to the dark at 22 °C and harvested at the time points indicated in Table 1 as described in the following. Three quarters of each plasmodium were harvested with a small glass spoon (Roth, Karlsruhe, FRG), shock-frozen in liquid nitrogen and stored at −80 °C for RNA isolation. One quarter was maintained in the dark overnight to monitor the developmental decision (*i*.*e*. whether or not sporulation had occurred). Dark control plasmodia were treated identically except that the light pulse was omitted. All manipulations were done under sterile conditions and under dim green safe light.

### Preparation of total RNA and RNAseq

For extraction of total RNA, the material obtained from a single plasmodium (approximately 75 mg fresh weight) was used. Samples were homogenized quickly at room temperature for 10 seconds in 3 ml peqGOLD TriFast (PeqLab; Nr 30–2020) using a glass potter and incubated for further homogenization for one minute at 50 °C. RNA was extracted with phenol/CHCl_3_ using 15 ml Phase Trap tubes according to manufacturer’s instructions (PeqLab; Nr 30-0150A-01). Afterwards the RNA was precipitated with ethanol in the presence of 0.3 M sodium acetate^32^. Total RNA was washed in 70% ethanol and re-dissolved in a final volume of 180 µL of RNase and DNase free water (T143.2, Roth, Karlsruhe, Germany).

Synthesis and amplification of cDNA for Illumina sequencing was performed as a commercial service at vertis Biotechnologie (Freising-Weihenstephan, Germany) as previously described^21^. Briefly, poly-A^+^ RNA was prepared from the total RNA samples and fragmented using ultrasound (4 pulses of 30 seconds at 4 °C). After treatment with tobacco acid pyrophosphatase (TAP), RNAs were re-phosphorylated with a polynucleotide kinase (PNK). The RNA fragments were then poly(A)-tailed using a poly(A) polymerase, followed by the ligation of a RNA adapter to the 5′ ends. First-strand cDNA synthesis was carried out with an oligo(dT)-adapter primer and the Moloney Murine Leukemia Virus (M-MLV) reverse transcriptase. The obtained cDNAs were amplified by PCR to about 30 ng/μl using a high fidelity DNA polymerase, and the PCR products were purified using the Agencourt AMPure XP kit (Beckman Coulter Genomics) and analyzed by capillary electrophoresis on a Shimadzu MultiNA microchip system. Barcoded cDNA libraries were prepared from samples of pooled or individual RNAs and subjected to Illumina sequencing (Table 1). Barcoding of RNA samples obtained from individual plasmodial cells should allow single cell analysis. cDNA samples were pooled in approximately equivalent amounts, and fragments in the range of 200 to 500 bp were fractionated from preparative agarose gels for single-end sequencing using the Illumina HiSeq. 2000 system^33^.

### Bioinformatic Analyses

In order to create a useful resource of characterized transcripts for future investigations, domain search and UniProt similarity search results for the reference transcriptome were combined into a common table. This table contains one or more entries per transcript according to the number of hits that were obtained in the domain search. Later on, information obtained for the differential regulation of each transcript was added to the corresponding entries of this table. From the comprehensive table (Supplementary Table 1), information about the domain composition, about the similarity to proteins in the UniProt database, and about the differential regulation during sporulation can be retrived for *all* transcripts of the reference transcriptome irrespective of whether or not a transcript is differentially regulated. For example, the domain assignments to those transcripts that are differentially regulated but do not have UniProt annotations can be easily retrieved. In parallel, the search results for similarity to *D*. *discoideum* genes were combined with the results on differential regulation (Supplementary Table S2).

## Electronic supplementary material

Supplementary Information

Supplementary Table 1

Supplementary Table 2

Supplementary Table 3

Supplementary Table 4

Supplementary Table 5
