# Supplementary material for: Transcriptome reprogramming during developmental switching in Physarum polycephalum involves extensive remodeling of intracellular signaling networks
Source: Sci Rep. 2017 Sep 26;7:12304. doi: 10.1038/s41598-017-12250-5 (PMC5615045; doi:10.1038/s41598-017-12250-5)
Supplement: Supplementary file 1 — Supplementary Information [file 41598_2017_12250_MOESM1_ESM.pdf]

Transcriptome reprogramming during developmental switching in *Physarum polycephalum* involves extensive remodeling of intracellular signaling networks

Gernot Glöckner and Wolfgang Marwan

Supplementray Information

Supplementary Tables	Description of Contents
Supplementary Table 1	All transcripts of the *P. polycephalum* reference transcriptome, no matter whether or not functionally regulated, queried against pfam and Prosite domain databases. The column „Regulation“ indicates the time period of maximal differential regulation of a *P.p.* transcript, nsr indicates no significant regulation. Each *P.p*. transcript may be represented by multiple entries if it gave hits to more than one domain.
Supplementary Table 2	All transcripts of the *P. polycephalum* reference transcriptome, no matter whether or not functionally regulated, queried against *Dictyostelium discoideum* genes. UniProt identifiers refer to *P.p.* transcripts. The differential regulation of *P.p.* transcripts is given as in Supplementary Tables 1 and 3.
Supplementary Table 3	Differentially regulated *P. polycephalum* transcripts with log2-fold change, p-value, and the time interval of maximal differential regulation, BLASTed against the Uniprot database. Abs_Log2_FC gives the absolute values of the Log2-fold changes.
Supplementary Table 4	Manual assignment of differentially regulated *P. polycephalum* transcripts functional according to the functional description of homologs in the Uniprot database. The table of contents (sheet 1) lists the groups of proteins to which the transcripts were assigned in the form of hyperlinks to the respective table.
Supplementary Table 5	Social amoebae core set of developmental genes BLASTed against the *P. polycephalum* transcriptome. Panel „ALL“ lists all social amoebae developmental genes out of the core set that gave hits to *P. polycephalum* transcripts. Panel „Regulated in Physarum“ lists only those genes that matched to differentially regulated *P. polycephalum* transcripts. Column B indicates, with which method the gene was defined as member of the core set (A, B, and C refer to ref 20 in the main text, and DE to ref 18 in the main text).
